# A Broadly Flavivirus Cross-Neutralizing Monoclonal Antibody that Recognizes a Novel Epitope within the Fusion Loop of E Protein

**DOI:** 10.1371/journal.pone.0016059

**Published:** 2011-01-11

**Authors:** Yong-Qiang Deng, Jian-Xin Dai, Guang-Hui Ji, Tao Jiang, Hua-Jing Wang, Hai-ou Yang, Weng-Long Tan, Ran Liu, Man Yu, Bao-Xue Ge, Qing-Yu Zhu, E-De Qin, Ya-Jun Guo, Cheng-Feng Qin

**Affiliations:** 1 State Key Laboratory of Pathogen and Biosecurity, Beijing Institute of Microbiology and Epidemiology, Beijing, China; 2 International Joint Cancer Institute, Second Military Medical University, Shanghai, China; 3 State Key Laboratory of Antibody Medicine and Targeted Therapy, Shanghai, China; 4 Institute of Health Sciences, Shanghai Institutes for Biological Sciences, Chinese Academy of Sciences/Shanghai JiaoTong University School of Medicine, Shanghai, China; Hallym University, Republic of Korea

## Abstract

Flaviviruses are a group of human pathogenic, enveloped RNA viruses that includes dengue (DENV), yellow fever (YFV), West Nile (WNV), and Japanese encephalitis (JEV) viruses. Cross-reactive antibodies against Flavivirus have been described, but most of them are generally weakly neutralizing. In this study, a novel monoclonal antibody, designated mAb 2A10G6, was determined to have broad cross-reactivity with DENV 1–4, YFV, WNV, JEV, and TBEV. Phage-display biopanning and structure modeling mapped 2A10G6 to a new epitope within the highly conserved flavivirus fusion loop peptide, the ^98^DRXW^101^ motif. Moreover, *in vitro* and *in vivo* experiments demonstrated that 2A10G6 potently neutralizes DENV 1–4, YFV, and WNV and confers protection from lethal challenge with DENV 1–4 and WNV in murine model. Furthermore, functional studies revealed that 2A10G6 blocks infection at a step after viral attachment. These results define a novel broadly flavivirus cross-reactive mAb with highly neutralizing activity that can be further developed as a therapeutic agent against severe flavivirus infections in humans.

## Introduction

Arthropod-borne flaviviruses, including dengue (DENV), Japanese encephalitis (JEV), West Nile (WNV), yellow fever (YFV) and tick-borne encephalitis viruses (TBEV), are related important human pathogens that cause severe hemorrhagic and encephalitic diseases of global impact [1], [2]. The mature flavivirus virion is spherical and enveloped, with a single-stranded, positive-sense RNA genome. Although the characteristics of these viruses are well-defined, no specific antiviral drugs are currently approved for clinical use against flavivirus infections. In viral diseases for which a specific therapy is not yet available, antibody-based therapy represents a promising alternative strategy. Neutralizing antibodies have been shown to be effective in animal models, both as prophylactics and as treatments for flavivirus infections [3]–[6]. Two humanized monoclonal antibodies (hE16 and CR4373) against WNV are in clinical trials [7].

Most neutralizing antibodies recognize the flaviviral envelope protein (E), which is the major glycoprotein on the surface of virions that plays a central role in receptor binding and membrane fusion. X-ray crystal structures have revealed that the E protein of flaviviruses has three domains, DI, DII and DIII [8]–[9]. Most potent type-specific and subcomplex-reactive neutralizing monoclonal antibodies (mAbs) mainly recognize the epitopes on DIII [10]–[14], which has been implicated in receptor binding. DII is formed from two extended loops that project from DI and contains a highly conserved fusion loop at its tip, i.e., amino acid residues 98–110, that interacts with the membranes of the target cell during fusion. Flavivirus cross-reactive mAbs have been reported, most of them directed against DII, with variable and less neutralizing profiles [15]–[18]. To date, only a few neutralizing mAbs against flaviviruses have been mapped to the fusion loop. Consequently, the precise antigenic structures of fusion loops and their functions in the protective immune response and pathogenesis remain poorly understood.

In this study, a novel flavivirus cross-reactive mAb, 2A10G6, directed against the highly conserved fusion loop, was generated and characterized. *In vitro* and *in vivo* neutralizing profiles of mAb 2A10G6 suggest that it is an ideal candidate for treating severe flavivirus infections.

## Materials and Methods

### Ethics Statements

All animal experimental procedures were carried out in strict accordance with the guidelines of the Animal Experiment Committee of the State Key Laboratory of Pathogen and Biosecurity, Ministry of Science and Technology of the People's Republic of China, and were approved by the Animal Experiment Committee of the State Key Laboratory of Pathogen and Biosecurity.

### Cells and viruses

BHK21 cells were maintained in Dulbecco's Modified Essential Medium supplemented with 10% fetal bovine serum (FBS) (ExCell). Mosquito C6/36, mouse myeloma SP2/0 and hybridoma cells were maintained in RPMI 1640 medium supplemented with 10–20% FBS. All cells were maintained in a 5% CO_2_ incubator at 37°C, except for the C6/36 cells, which were maintained at 28°C.

The flavivirus strains used in this study were DENV1-128, DENV2-43, DENV3-80-2, DENV4-B5, YFV-17D, WNV-chin01, JEV-BJ-01, and TBEV-Senzhang. Viruses were prepared from culture supernatants of infected mosquito C6/36 cells or infected suckling mouse brain suspensions.

### mAb preparations

Six-week-old female BALB/c mice were subcutaneously immunized twice at 3-week intervals with 400 µl of heat-inactivated DENV2 emulsified in Freund's complete or incomplete adjuvant (Sigma). Three days after a final immunization with virus antigens (infected sulking mouse brain suspensions) alone, spleen cells from the mice and mouse myeloma SP2/0 cells were fused and maintained according to the standard procedure [19]. Hybridomas were screened for secretion of anti-DENV2 specific mAbs using an indirect immunofluorescence assay, and then E-specific mAbs were identified by immunostaining of BHK21 cells transfected with plasmids expressing the prM-E protein. The hybridoma producing mAb 2A10G6 (IgG1) was cloned twice via limiting dilutions of the cells. mAb 2A10G6 was purified from mouse ascites using protein A affinity columns (GE).

### Indirect immunofluorescence assay

Indirect immunofluorescence assays (IFA) were performed as follows. Briefly, BHK21 cells were infected with various flavivirus strains and fixed with ice-cold acetone. Cells were incubated with a 100-fold dilution of mAb 2A10G6 or normal mouse serum. After 60 min of incubation at 37°C, cells were washed three times with phosphate-buffered saline (PBS). Cells were then treated with a 200-fold dilution of FITC-conjugated anti–mouse IgG (KPL) in 0.02% (w/v) Evans blue for 30 min at 37°C and rinsed with PBS. After five washes in PBS, positive cells were detected using a fluorescent microscope.

### Western blot analysis

The DENV2 prME proteins were expressed in eukaryotic systems following the manufacturer's instructions and as previously described [20], [21]. Briefly, pcDNA3.1-based plasmids encoding the DENV2 prME genes were constructed. Then, BHK21 cells were transiently transfected with recombinant plasmids encoding the prME protein or with empty pcDNA3.1 (control) using Lipofectamine 2000 reagent (Invitrogen).

At 48 h after transfection, cultured cells were lysed and analyzed by western blotting. Briefly, cell lysates containing the prME protein were separated using 12% SDS-PAGE and transferred to nitrocellulose membranes. The membranes were blocked with PBST containing 5% skim milk and then reacted with the appropriate mAb 2A10G6 at room temperature (RT) for 1 h. Subsequently, membranes were washed three times with 0.1% Tween 20/Tris-buffered saline (TBST) and incubated with alkaline phosphatase–conjugated goat anti-mouse secondary antibody (1∶1,000). Following incubation for 1 h at RT, the membranes were washed three times with TBST and developed with NBT/BCIP (KPL).

### Indirect enzyme-linked immunosorbent assay

The DENV2 DI–DII or DIII was expressed in *Escherichia coli* as a fusion protein with thioredoxin following the manufacturer's instructions and as previously described [22]. Briefly, the pET32a-based plasmid (Novagen) encoding DI–DII, residues 1 to 297, or DIII, residues 298 to 400, of the DENV2-43 strain was constructed. Then the recombinant DI–DII or DIII fusion protein was expressed in the *E. coli* strain BL21 (DE3) after induction with 1 mM isopropyl-β-D-thiogalactopyranoside (IPTG). The crude cell debris was denatured using 8 M urea, refolded by step dialysis and purified over a nickel-chelated column (Pierce).

An indirect ELISA was used to investigate the specificity of mAb 2A10G6 for the viral antigen. Briefly, 96-well microtiter plates were coated overnight at 4°C with recombinant DI–DII or DIII protein (1 µg/ml) in a pH 9.6 carbonate buffer. Plates were washed three times with PBST and blocked for 2 h at 37°C with PBST containing 5% skim milk. Subsequently, plates were rinsed five times in PBST and then incubated with different concentrations of 2A10G6 in triplicate for 1 h at RT. Plates were washed five times and then incubated with peroxidase-conjugated goat anti–mouse IgG (1∶5000) (KPL) for 1 h at RT. Plates were washed five times and then sequentially incubated with TMB substrate (Promega). The reaction was stopped by the addition of 2 N H_2_SO_4_ to the medium, and emission (450 nm) was read using a microplate reader (Beckman).

### Phage-display biopanning assay

Phage-display experiments were performed as previously described [23], [24]. Briefly, 100 µg/ml anti-DENV antibody 2A10G6 was immobilized on 96-well plate. Phages [1.5×10^11^ plaque-forming units (PFU)] from the Ph.D.-12 Phage Display Peptide Library (New England Biolabs) were incubated at 37°C for 1 h with immobilized 2A10G6. The wells were washed five times with TBST. Then, the phages that bound 2A10G6 were amplified by direct infection of *E. coli* ER2738. The amplified phages were purified by precipitation with 20% PEG 8000/2.5 M NaCl and used in the next cycle. Three rounds of selection were routinely performed. The immunopositive phage clones were screened by ELISA and further characterized by DNA sequencing.

### Sequence alignment and molecular modeling

Nucleotide and deduced amino acid sequences encoding the E genes of the flaviviruses tested were obtained from GenBank. The NCBI accession numbers are FJ176780 (DENV1-128), AF204178 (DENV2-43), AF317645 (DENV3-80-2), AF289029 (DENV4-B5), X03700 (YFV-17D), AY490240 (WNV-chin01), L48961 (JEV-BJ01) and AY174188 (TBEV-Senzhang). Sequence analysis was performed using MEGALIGN in the Lasergene software (DNAstar).

Based on the crystal structures of DENV-2 E protein (PDB code: 1OK8), the probable epitope of 2A10G6 was analyzed using Discovery studio 2.0 (Accelrys). The location and structure of the 2A10G6 epitope in the three known E protein crystal structures of WNV and TBEV (PDB codes: 2I69 and 1SVB, respectively) were also analyzed.

### Neutralization assay

Five-fold serial dilutions of 2A10G6 were added to approximately 200 PFU of a variety of flavivirus strains and incubated at 37°C for 60 min. Then, the mixture was added to BHK21 cell monolayers in a 6-well plate in duplicate and incubated for 60 min at 37°C. The supernatant was removed, and 3 ml of 1.0% (w/v) LMP agarose (Promega) in DMEM plus 4% (v/v) FBS was layered onto the infected cells. After further incubation at 37°C for 4 to 7 days, the wells were stained with 1% (w/v) crystal violet dissolved in 4% (v/v) formaldehyde to visualize the plaques. The percentage of plaque reduction was calculated as previously described [13].

### Animal experiments

DENV 1–4 were prepared from infected suckling mice brain suspensions. Viruses (10^4^ PFU/ml) were mixed with an equal volume of serial dilutions of 2A10G6 and incubated for 60 min at 37°C. Then the mAb-virus mixture was injected intracerebrally into 1-day-old suckling mice. The control group only received PBS diluent. The animals were monitored daily for clinical signs of infection, including ruffled hair, a hunched back, paralysis, and death, for 3 weeks. For antibody protection against West Nile virus challenge, groups of 4-week-old female Balb/C mice were treated by intraperitoneal injection with 200 µg of 2A10G6 or PBS one day before or after challenge with 40 PFU of WNV, respectively. Mice were then monitored daily for 15 days for mortality. Protection significance was evaluated using the log-rank test and compared to the PBS controls.

### Cell-binding assay

Individual mAbs (50 µg/ml of 2H11, 2A10G6, or 2B8) or BSA was incubated with 10^3^ PFU of DENV2 for 60 min at 4°C. The virus–mAb mixtures were then added to BHK21 cells in 12-well plates for 60 min on ice. Unbound virus was removed after three washes with prechilled PBS. Total RNA was extracted from infected cells using Trizol reagent (Invitrogen), and viral RNA was quantified by real-time RT-PCR as previously described [25].

### Pre- and post-adsorption inhibition assay

Neutralization of DENV2 before or after adsorption to BHK21 cells was performed using 10^2^ PFU of DENV2 and serial dilutions of 2A10G6 or 2B8 essentially as described previously [26]. In the post-adsorption assay, DENV2 firstly were added to BHK21 cells for 60 min at 4°C, then the mAb was added and incubated for additional 60 min at 4°C. In the pre-adsorption assay, the mAb was firstly incubated with BHK21 cells for 60 min at 4°C before DENV was added. After three times washes with PBS, the PRNT protocol was followed as described above.

## Results

### Characterization of the flavivirus cross-reactive mAb

**Figure 1:**
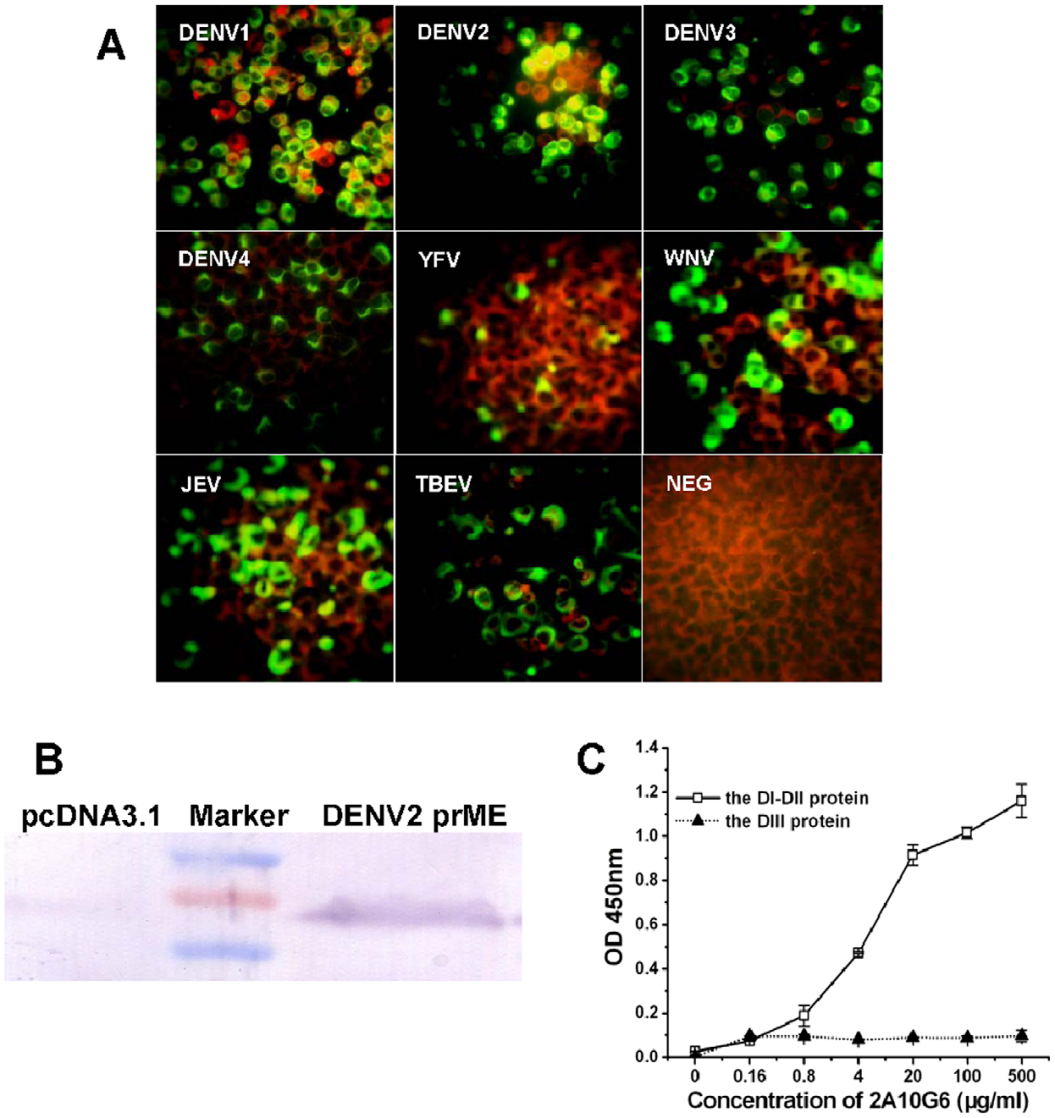
Characterization of mAb 2A10G6 *in vitro*. **A.** Cross-reactivity of 2A10G6 with four DENV serotypes and four other representative flaviviruses determined by indirect immunofluorescence analysis. BHK21 cells were infected separately with DENV1–4, YFV, WNV, JEV, or TBEV. Three to 5 days after infection, cells were fixed and analyzed by IFA with 2A10G6. Uninfected cells were run simultaneously as negative controls. **B.** The specificity of 2A10G6 for the DENV2 prME protein. BHK21 cells were transiently transfected with the recombinant plasmid encoding the prME protein or with a control vector, empty pcDNA3.1. At 48 h after transfection, cultured cells were lysed and analyzed by western blotting with 2A10G6. **C.** The specificity of 2A10G6 for the DENV2 DI–DII peptide. The DENV2 DI–DII peptide was expressed in *E. coli* as a fusion protein with thioredoxin, and binding of different concentrations of 2A10G6 with DENV2 antigen and recombinant DI–DII peptide was detected by ELISA.

MAb 2A10G6 was originally identified as a DENV2-specific mAb by indirect immunofluorescence assay (**Fig. 1A**). To first determine whether the viral surface glycoprotein is recognized by 2A10G6, the reactivity of 2A10G6 to prME protein was examined by western blotting. As expected, 2A10G6 reacted with BHK21 cells transfected with the recombinant plasmid expressing the DENV2 prME protein, suggesting that 2A10G6 is directed against the DENV2 prME protein (**Fig. 1B**). Further, to determine the domain of the DENV2 E protein responsible for binding, an indirect ELISA was used to detect the specific binding of 2A10G6 with the recombinant DI–DII or DIII of the DENV2 E protein. We found that 2A10G6 only reacted with DI–II of the DENV E protein (**Fig. 1C**). Subsequently, the cross-reactivity of 2A10G6 was tested by IFA using other DENV serotypes and related flaviviruses. We detected cross-reaction of 2A10G6 with DENV 1–4, YFV, WNV, JEV, and TBEV (**Fig. 1A**). Therefore, a broadly flavivirus cross-reactive mAb 2A10G6 was identified.

### The specific site recognized by 2A10G6 is the ^98^DRXW^101^ motif, located at the tip of the fusion loop of E protein

To map the epitope determinants of 2A10G6, a standard phage-display technique was performed. After three rounds of panning, 94 positive phage clones were identified and subjected to DNA sequence analysis. The results showed that 86 of the positive clones displayed 12 amino acid residues in common, i.e., SNFFDRTWPKLT; the other three clones displayed YNFFDRTWPKLT, SHRQHETDRNWP, or NYPEDFFQRTWP. Specifically, seven residues, FFDRTWP, were conserved in these immune-positive phage clones, and the other five residues were random. Thus, the binding sites of 2A10G6 were deduced to be FFDRTWP.

**Figure 2:**
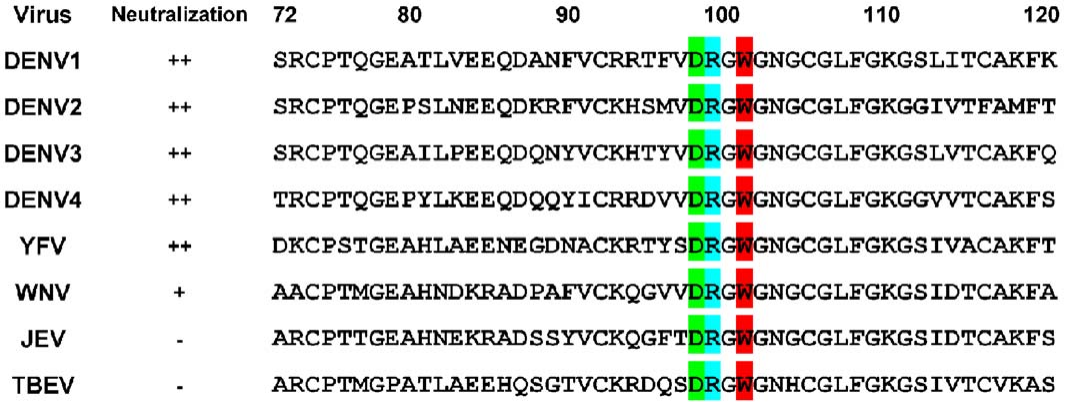
Comparison of the amino acid sequences of flavivirus E proteins. Amino acids at positions 72 to 120 are shown. Boxed residues indicate that positions 98 (green), 99 (blue), and 101 (red) were highly conserved among the flaviviruses examined and constitute a conformational epitope.

We next compared this peptide with the amino acid sequences of E proteins of DENV1–4, YFV, WNV, JEV, and TBEV and found that all these flavivirus strains shared the same amino acids at positions 98 (D), 99 (R), and 101 (W) within the highly conserved N-terminal fusion loop peptide of the E protein DII (**Fig. 2**). To verify that the ^98^DRXW^101^ motif was recognized by 2A10G6, a peptide (MVDRGWG) corresponding to the amino acids of the DENV2 E protein was synthesized, and the reactivity of 2A10G6 to the synthetic peptide was determined by ELISA. The results showed that 2A10G6 specifically reacted with the synthetic peptide MVDRGWG (data not shown), suggesting that this region containing the three common residues constitutes a mimotope of the 2A10G6 epitope.

**Figure 3:**
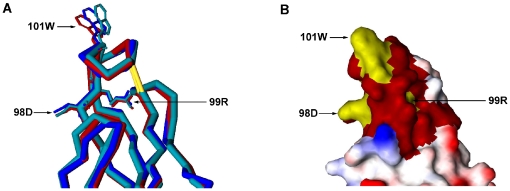
Structural mapping of the 2A10G6 epitope. **A.** the structure of the 2A10G6 epitope in the crystal structure of DENV2 E protein. Residue numbering is based on the E gene sequences. The regions in red are thought to be structurally near these three residues. **B.** Three-dimensional comparisons of the 2A10G6 epitope in the three known crystal structures. DENV2, WNV, and TBEV E proteins are in shades of red, blue, and cyan, respectively.

Furthermore, three-dimensional structural analysis showed that the ^98^DRXW^101^ motif was located at the tip of the fusion loop of the DENV2 E protein (**Fig. 3A**). The structure of the 2A10G6 epitope in the three known E protein structures was compared (**Fig. 3B**). The comparison indicated that the conformational epitope formed by the three residues D, R, and W was similar among flaviviruses (**Fig. 3B**). Together, these findings suggest that 2A10G6 recognizes a new epitope within the flavivirus fusion loop.

### Neutralizing and protective profiles of 2A10G6

**Figure 4:**
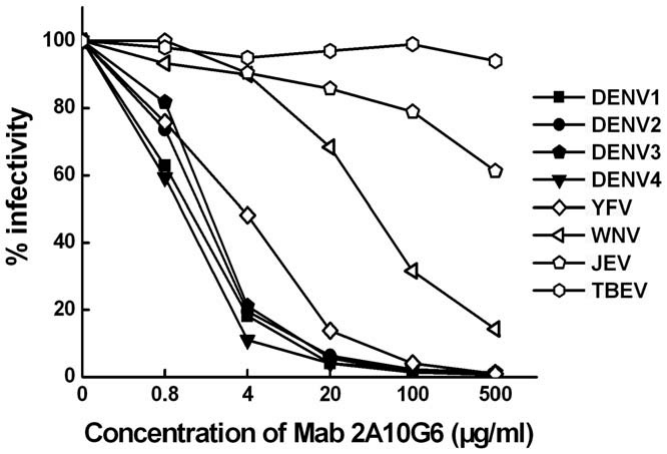
Neutralization activity of 2A10G6 to various flavivirus strains. Viruses were mixed with 2A10G6 at different concentrations. Neutralization activities were evaluated by plaque reduction assays using BHK21 cells.

To investigate the neutralizing and protective potential of mAb 2A10G6, the neutralizing activities of 2A10G6 for various flavivirus strains were assessed using a standard plaque reduction neutralization assay. 2A10G6 exhibited high neutralizing activity against DENV 1–4 and YFV, with 50% plaque reduction neutralization titers (PRNT_50_) of 2, 1.5, 2.1, 1.8, and 3.6 µg/ml, respectively. WNV was also neutralized by 2A10G6 at a PRNT_50_ of 46 µg/ml. Although IFA results (**Fig. 1**) showed that 2A10G6 can also recognize JEV and TBEV, no neutralizing activity of 2A10G6 was observed for JEV and TBEV (**Fig. 4**). These results indicate that 2A10G6 can potently neutralize DENV 1–4, YFV, and WNV.

**Figure 5:**
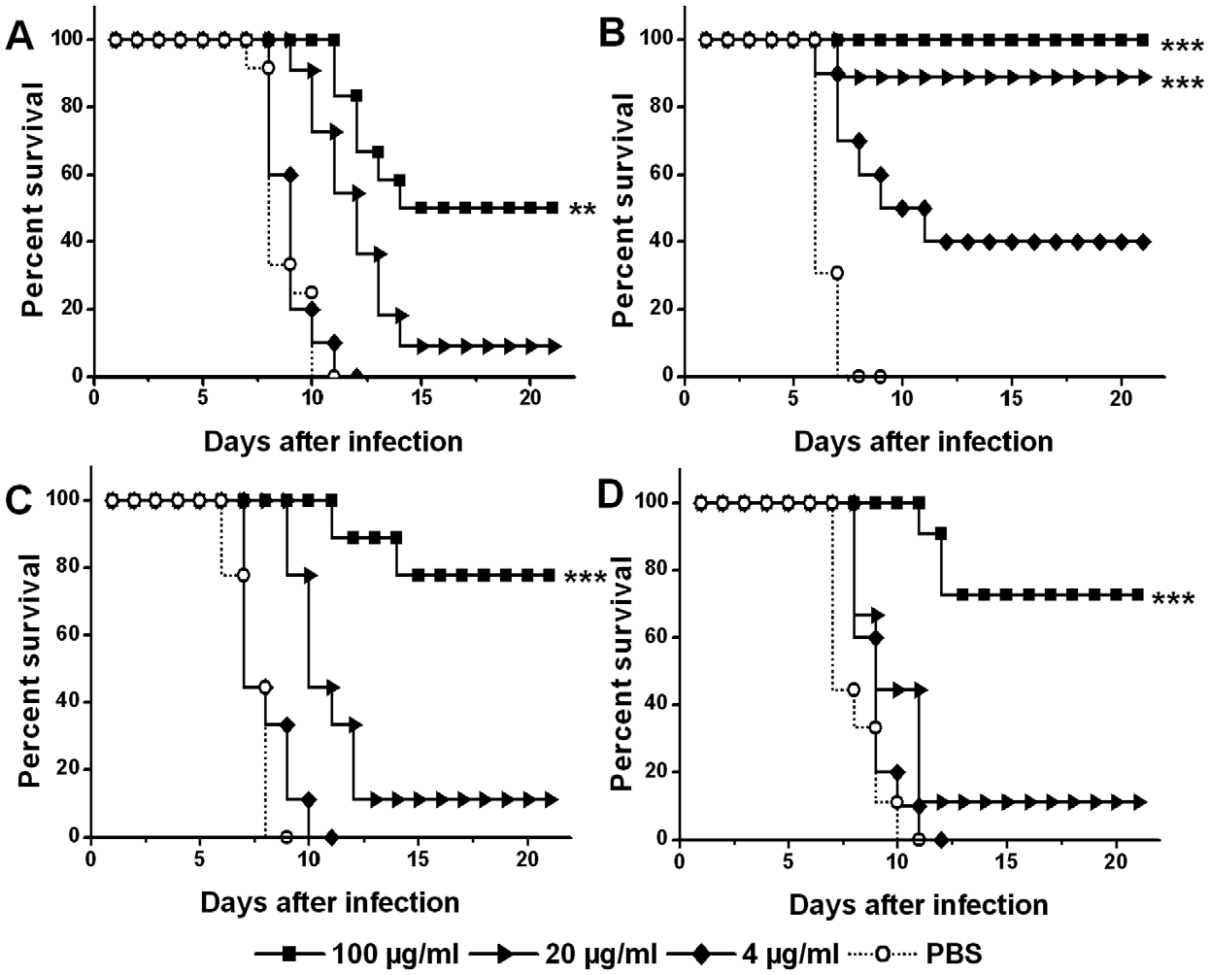
Protection of 2A10G6 against DENV 1-4 in suckling mice. 2A10G6 at 100, 20, or 4 µg/ml was incubated with 10^4^ PFU/ml of DENV 1–4 and inoculated into brains of suckling mice. Mortality was the outcome measured. Viruses with PBS were included as a negative control. The number of animals for each antibody dose ranged from 9 to 12. Kaplan–Meier survival curves were analyzed by the log-rank test and compared to curves of the PBS controls. Significant differences are indicated by asterisks (*** p<0.001, ** p<0.01 and * p<0.05). A, B, C, and D represent DENV1, DENV2, DENV3, and DENV4 infection, respectively.

To characterize the protection profile of 2A10G6 *in vivo*, an established suckling model was employed to analyze the protective efficacy of 2A10G6 against lethal DENV 1–4 infection. The control mice developed typical neurological symptoms and died at 6 to 7 days post-infection. As shown in Fig. 5, 2A10G6 treatment showed protection against DENV 1–4 in a dose-dependent manner. Especially, treatment of 100 µg/ml 2A10G6 conferred full protection against lethal DENV2 challenge, and 20 µg/ml and 4 µg/ml 2A10G6 protected 89% and 40% of infected mice from lethal challenge, respectively (**Fig. 5B**). For infection with DENV1, 3, and 4, use of 100 µg/ml 2A10G6 conferred partial protection, and 53%, 77%, and 73% of the infected mice survived after challenge, respectively. Statistical analysis using the log-rank test showed that survival rate of 2A10G6-treated mice were significantly higher than that of PBS controls, indicating that 2A10G6 confers protection against DENV 1–4 infection *in vivo*.

**Figure 6:**
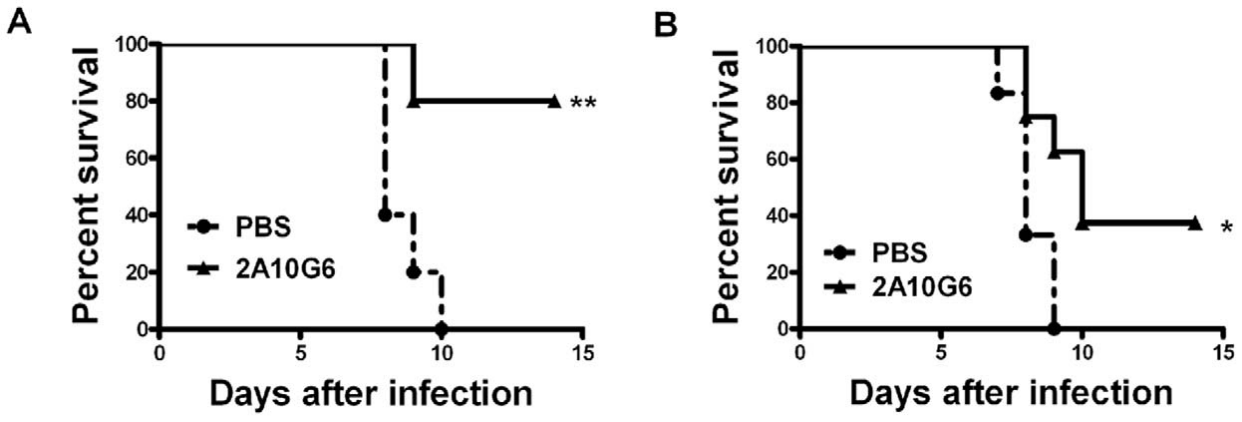
Protetcion of 2A10G6 against WNV in mice. Group of 4-week-old Balb/C mice were administrated with 200 µg of 2A10G6 one day before (A) or after (B) challenge with 40 PFU of WNV. PBS was included as a negative control. Kaplan–Meier survival curves were analyzed by the log-rank test and compared to curves of the PBS controls. Significant differences are indicated by asterisks (** p<0.01 and * p<0.05).

Additionally, the protection profile of 2A10G6 against WNV was also investigated in mice. The results (**Fig. 6**) showed that prophylactic administration with a single dose of 200 µg of 2A10G6 conferred 80% protection in mice. Most importantly, 3 of 8 (37.5%) mice survived when 2A10G6 was administered one day after WNV challenge. Log rank analysis showed that a significant difference among groups between 2A10G6 and PBS control. There results strongly indicated the therapeutic potential of 2A10G6 in antibody based therapy against severe flavivirus infections.

### Mechanism of 2A10G6-mediated neutralization

**Figure 7:**
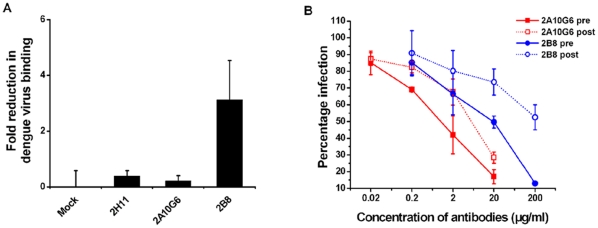
Mechanism of 2A10G6-mediated neutralization. **A.** Dengue virus binding to BHK21 cells. The DIII-specific neutralizing mAb 2B8 blocked cellular attachment significantly more than the DI–DII–specific neutralizing mAb 2A10G6 or controls, including no antibody (BSA) and the non-neutralizing mAb 2H11. Fold reductions are reported with standard deviations as the average of four independent experiments. **B.** Pre- and post-adsorption inhibition assays. Pre-binding of DENV2 with 2A10G6 or 2B8 significantly protected against infection (solid lines) (Pre). In contrast, 2A10G6 but not 2B8 inhibited infection when added after virus binding (dotted lines) (Post). Infection percentages at the different antibody concentrations are shown with standard deviations.

To further define the mechanism of 2A10G6-mediated neutralization of dengue virus, binding inhibition assays were performed with BHK21 cells. Two DIII-specific mAbs with (2B8) or without (2H11) neutralizing activity were used as controls. As expected, 2B8 significantly (P<0.01) inhibited virus binding by 3.1-fold, whereas BSA protein or 2H11 failed to inhibit virus binding (**Fig. 7A**). Although 2A10G6 can bind to DENV E protein (**Fig. 1**), 2A10G6 did not inhibit virus binding, suggesting that the activity of 2A10G6 may not involve the blockade of cell attachment.

Because 2A10G6 supposedly recognizes the fusion loop, it might inhibit a step after cellular attachment. Therefore, we performed a pre- and post-adsorption inhibition assay. 2A10G6 or 2B8 were incubated with DENV2 before or after its binding to a monolayer of BHK21 cells, and virus titer was measured by a plaque reduction assay. As expected, both 2A10G6 and 2B8 neutralized infection when applied before the binding of the virus. However, 2A10G6, but not 2B8, inhibited DENV infection when added after virus adsorption to the cell surface (**Fig. 7B**). These results indicate that 2A10G6-mediated neutralization occurs primarily after dengue virus cellular attachment.

## Discussion

In the present study, we generated and characterized a novel broadly flavivirus cross-reactive mAb, 2A10G6, that strongly neutralized several flavivirus infections *in vitro* and protected mice against lethal DENV1–4 and WNV infection *in vivo*. Furthermore, 2A10G6 recognized the highly conserved fusion loop peptide ^98^DRXW^101^. Functional experiments suggested that 2A10G6 neutralized viral infectivity at a post-attachment step in the viral life cycle.

During neutralization *in vitro*, 2A10G6 displayed potent neutralizing activities against DENV1–4, YF, and WN viruses but was unable to neutralize JE and TBE viruses. Several flavivirus cross-reactive mAbs have been reported to have neutralizing activities. For example, a humanized mAb, 1A5, derived from a chimpanzee has similar cross-reactivities but different neutralizing activities for DENV1–4 [27]. Another study showed that mAb against the WNV E protein cross-reacted with DENV1–4 but only neutralized DENV2 and DENV4 [28]. Our results indicate that 2A10G6 is a more broad-spectrum neutralizing mAb in comparison with other reported mAbs. Consistent with neutralization *in vitro*, 2A10G6 had potent protective efficacies against DENV1–4 and WNV *in vivo*, although these efficacies were somewhat different.

We mapped the epitope of 2A10G6 to the ^98^DRXW^101^ motif in the highly conserved N-terminal fusion loop at the tip of DII in the E protein. Three-dimensional structural analyses based on the crystal structures of DENV2 E protein revealed that the three amino acids D98, R99, and W101 are exposed on the virion surface and constitute a novel conformational epitope. These three amino acids are highly conserved among flaviviruses, including DENV 1–4, WNV, JEV, YFV, and TBEV (**Fig. 3**). Previous studies reported that several mAbs recognize a set of overlapping epitopes that form an antigenic site on the fusion loop surface of DII. For instance, mAb E53 recognizes an epitope including residues within the fusion loop and the adjacent bc-loop in DII of E protein [17], [29]. Similarly, the epitope of Mab11 was mapped by yeast surface display to include residues in the fusion loop (W101, G104, and G106) [30]. Furthermore, W101, L107, and F108 were also found to be shared by several different but overlapping epitopes recognized by the predominantly cross-reactive anti-E antibodies present in polyclonal sera from patients with dengue primary infection [31], [32]. In addition, our structural analysis indicated that the binding sites of 2A10G6 might involve other surface-exposed residues that are spatially close to these three residues (the red region in **Fig. 3A**). Thus, the 2A10G6 epitopes represent novel antigenic sites for a potently cross-neutralizing antibody on the N-terminal fusion loop.

Among the known structures of flavivirus E protein, the backbones of the fusion loop are highly similar. Nine residues in this region constitute the largest surface-exposed cluster in the post-fusion structure [9], [33]. Therefore, the 2A10G6 epitope might determine membrane fusion and cross-neutralizing and may be a good target for broad-spectrum therapeutic antibodies or other antiviral therapeutics. However, like many fusion loop–specific neutralizing mAbs, 2A10G6 showed different neutralizing activities against different strains, suggesting the existence of subtle structural differences in this region around the 2A10G6 epitope that perhaps involve the polarities and side chain orientations of structurally neighboring but less-conserved amino acids. In addition, the exposed epitopes available for antibody recognition in the structural transition process are likely to vary in different flaviviruses [12], [34], giving mAb 2A10G6 similar but distinct cross-neutralizing reactivity profiles.

Our experiments also showed that 2A10G6 did not significantly inhibit cellular attachment of DENV2 to BHK21 cells, but that it inhibited infection when added after virus binding, suggesting that 2A10G6 neutralizes viral infectivity at a post-attachment step in the viral life cycle. Generally, antibodies have the potential to neutralize the infectivity of flaviviruses by interfering with several steps of the virus entry pathway, including attachment, internalization and fusion, perhaps depending on the locations of their binding sites [6], [35]. Previous studies have shown that mAbs against the TBEV fusion loop were effective at blocking liposomal fusion [36]. However, E53 inhibited infection primarily by blocking viral attachment to some cells [17] or by blocking the transition from an immature to a mature structure [29].

Flavivirus infection can cause fever, encephalitis, hemorrhagic disease, flaccid paralysis and death in humans. In the last decade, the flaviviruses have re-emerged as aggressive human pathogens causing an increased number of infections worldwide [2]. Although much effort has been made to develop effective antiviral drugs, no specific therapies are currently available. DENV infection represents a major public health problem with an estimated 2.5 billion people at risk of infection in tropical and subtropical countries, mainly southeast and south Asia, Central and South America, and the Caribbean [37]. In additional, multiple flavivirus infections have been reported in the same areas, making early diagnosis and discrimination a substantial challenge. For example, JEV and DENV are both endemic in many Asian countries, whereas YFV and WNV are reported in Africa and South America [1]. To control the growing public health problem of flavivirus infection, new antiviral therapeutic strategies that provide potent and broadly cross-protective host immunity are therefore a global public health priority [7]. At present, human mAb–based “passive” immunotherapies for these viruses are at a very early stage of development, including for WNV [38]. Previous studies in mice have shown that passive transfer of either monoclonal or polyclonal antibodies can be protective against homologous or heterologous DENV challenge [27], [39]–[40]. For this reason, mAb 2A10G6 may further be humanized into a therapeutic antibody to treat DENV and other severe flavivirus infections.

In summary, we generated and characterized a broadly flavivirus cross-reactive mAb, 2A10G6, with *in vitro* and *in vivo* neutralizing activity, and demonstrated the potential therapeutic potential of humanized 2A10G6 for multivalent treatment against severe flavivirus infections.
